# Biological Control of Mosquito Vectors: Past, Present, and Future

**DOI:** 10.3390/insects7040052

**Published:** 2016-10-03

**Authors:** Giovanni Benelli, Claire L. Jeffries, Thomas Walker

**Affiliations:** 1 Insect Behaviour Group, Department of Agriculture, Food and Environment, University of Pisa, Pisa 56124, Italy; benelli.giovanni@gmail.com; 2 Department of Disease Control, London School of Hygiene and Tropical Medicine, London WC1E 7HT, UK; claire.jeffries@lshtm.ac.uk

**Keywords:** mosquito-borne diseases, arboviruses, boosted SIT (Sterile Insect Technique), copepods, larvivorous fishes, sex pheromones, sterile insect technique, sound traps, swarm manipulation, *Wolbachia* bacteria

## Abstract

Mosquitoes represent the major arthropod vectors of human disease worldwide transmitting malaria, lymphatic filariasis, and arboviruses such as dengue virus and Zika virus. Unfortunately, no treatment (in the form of vaccines or drugs) is available for most of these diseases and vector control is still the main form of prevention. The limitations of traditional insecticide-based strategies, particularly the development of insecticide resistance, have resulted in significant efforts to develop alternative eco-friendly methods. Biocontrol strategies aim to be sustainable and target a range of different mosquito species to reduce the current reliance on insecticide-based mosquito control. In this review, we outline non-insecticide based strategies that have been implemented or are currently being tested. We also highlight the use of mosquito behavioural knowledge that can be exploited for control strategies.

## 1. Introduction

**Figure 1:**
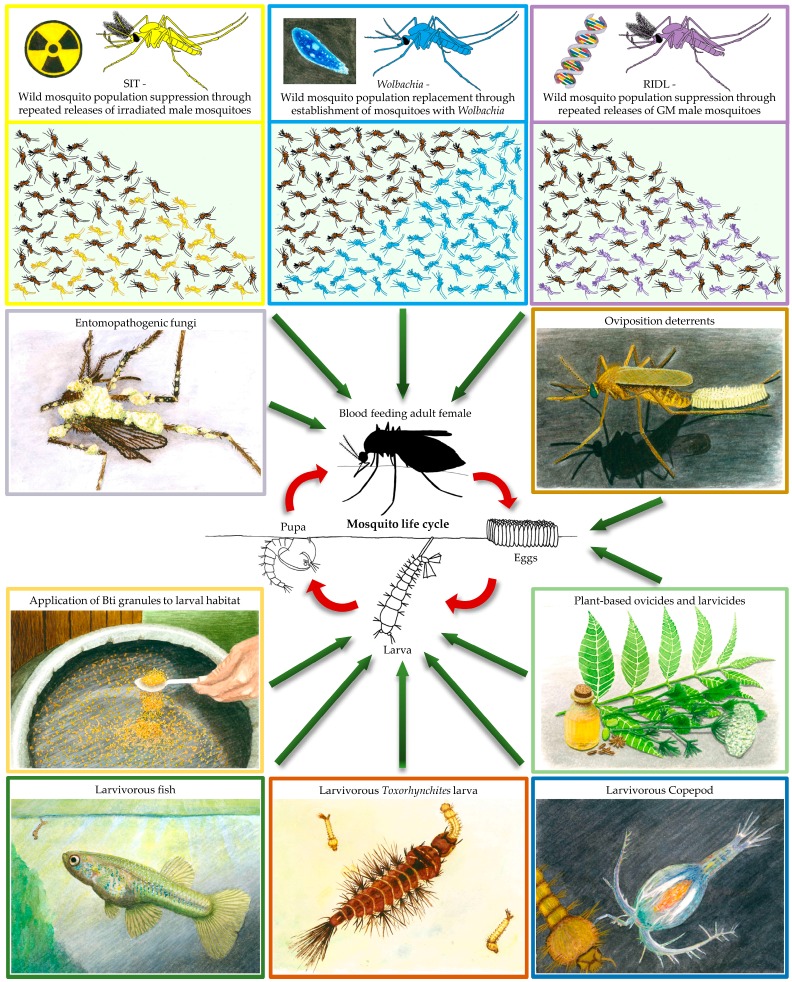
Mosquito biocontrol strategies targeting different stages of the mosquito lifecycle.

Vector control strategies have traditionally focused on killing mosquitoes using a variety of insecticides. Environmental management (through reduction or removal of mosquito breeding sites) has often been used alongside chemical or microbiological ovicides, larvicides, and pupicides [1,2,3,4] in areas where endemic mosquito-borne diseases occur. The use of synthetic insecticides has to be regulated given that the development of insecticide resistance is widespread [5,6,7,8,9] and that there is concern regarding the damage to the environment and effects on non-target organisms. The use of insecticides for mosquito control, including organophosphates, carbamates, and pyrethroids, can also have negative effects on human health. Personal protection against mosquito-borne diseases can involve the use of mosquito repellents such as *N,N*-diethyl-meta-toluamide (DEET), dimethyl phthalate (DMP), *N,N*-diethyl mendelic acid amide (DEM), as well as plant-borne molecules (reviewed by [10]), light-coloured clothes covering as much of the body as possible, and sleeping under mosquito nets. Insecticide-treated bednets have played a very important role in the reduction of *Plasmodium falciparum* infection prevalence in malaria endemic Sub-Saharan Africa, which has seen the incidence of clinical disease fall by 40% between 2000 and 2015 [11]. However, bednets are only effective against mosquitoes that bite during the night and concern is growing that insecticide resistance, particularly due to the most commonly used class of pyrethroids, could reverse this trend and lead to rising incidence of malaria and increased fatalities [12]. As insecticide resistance is now widespread in a number of mosquito species [6,8,9], there is a growing need for novel, cheap, and reliable mosquito control strategies [13,14,15]. In many countries where mosquito-borne diseases are endemic, the financial burden of insecticide-based vector control programs is also prohibitive to widespread use. Environmentally friendly alternatives have been explored to help reduce the selection pressure for insecticide resistance. These various biocontrol strategies target different stages of the mosquito lifecycle (Figure 1) with the aim of being safe for the environment and sustainable. These diverse biocontrol strategies include natural organisms that kill mosquitoes, exploiting mosquito behaviour to improve mosquito mortality, and releasing mosquitoes that are either sterile or unable to transmit disease.

## 2. Using Biocontrol to Kill Mosquitoes

### 2.1. Plant-Borne Mosquitocides, Repellents, and Oviposition Deterrents

The discovery of the plant-based drug artemisinin for malaria treatment [16] and the subsequent awarding of the Nobel prize in 2015 [17] highlights the importance of screening plants and fungi as sources of metabolites for parasitological and mosquitocidal properties. Notably, plant-borne molecules are often effective at a few parts per million (ppm) against *Aedes (Ae.)*, *Anopheles (An.)* and *Culex (Cx.)* young larval instars (see [4] and [18] for dedicated reviews on ovicides and larvicides, respectively). Currently, more than 80 plant species have been employed for the successful synthesis of nanomosquitocides, with particular reference to larvicidal purposes. On the other hand, studies on ovicidal and ovideterrent nanoformulates are limited [19]. Furthermore, botanicals can also be used as reducing and capping agents for the rapid synthesis of mosquitocidal nanoformulations [20], and can even be employed to prepare cheap repellents with low human toxicity [3]. Notably, much remains to be discovered about this fast-growing research area, with special reference to the following topics: (i) the chemical characterization and standardization of plant-borne botanicals used for nanobiosynthesis [13], (ii) the potential of plant-synthesized nanoparticles as mosquito ovicides and ovideterrents [21], (iii) the utility of industrial by-products of plant origin for biofabrication of nanomosquitocides (e.g., neem cake) [4], (iv) field evaluation of mosquitocidal properties of green nanoparticles against Culicidae [22,23], (v) the non-target effects and environmental fate of plant-synthesized nanoparticles used against mosquito vectors [20].

### 2.2. Mosquito Predators

Natural enemies feeding on mosquito larvae and pupae in aquatic environments can play an important role in reducing Culicidae populations [24,25,26]. Indeed, mosquito young instars are preyed upon by a large number of aquatic organisms including fish [21,27,28,29], amphibians [30,31], copepods [32,33], odonate young instars [34], water bugs [35,36,37,38], and even larvae of other mosquito species [39,40]. Biological control of mosquitoes using vertebrates has mostly focused on the role of larvivorous fish that consume the aquatic larval stage of mosquitoes [26]. Fish predation of mosquito larvae has been recorded in many habitats, from small plastic containers [41] to complex natural ecosystems, including coastal wetland environments [42]. Larvivorous fish have been demonstrated to be very effective at reducing mosquito larval populations in many parts of the world, and in a variety of habitats [25,27,43]. In particular, larvivorous fish belonging to the genus *Gambusia* and *Poecilia* (Poeciliidae) have been introduced in more than 60 countries for mosquito control purposes [27,28,44,45,46,47,48]. However, introduced larvivorous fish are often considered a threat to native aquatic fauna, including amphibians [49,50], highlighting the need to carefully consider the ecological cost of introducing predatory species intended to contribute to mosquito control.

A number of omnivorous copepods (small aquatic cyclopoid crustaceans) can prey on young mosquito larval stages [51]. Several species of copepods, such as *Cyclops vernalis*, *Megacyclops formosanus*, *Mesocyclops (M.) aspericornis*, *M. edax*, *M. guangxiensis*, *M. longisetus* and *M. thermocyclopoides*, have been reported as active predators of mosquito young instars [32,52,53,54,55,56,57,58]. Operationally, the use of copepod predators against mosquitoes in urban and semi-urban habitats is not expensive and requires minimal labour for colony maintenance, highlighting their easy and cheap potential as mass-reared biocontrol agents [59,60]. The largest and most successful application of copepods for mosquito control was carried out in Vietnam to target the principal vector of dengue virus (DENV), *Ae. aegypti* [61]. From an initial introduction of copepods into a village in northern Vietnam in 1993, *Ae. aegypti* was eradicated from large surrounding areas by 2000, and dengue transmission could not be detected. Copepod biocontrol for *Ae. aegypti* was still being actively undertaken by communities in Vietnam even after the official intervention had ceased [62,63]. However, there are limitations in terms of the specific mosquito species to which copepods can be efficiently applied, since the larval habitats of many mosquito species are not suitable for copepods [64].

The larvae of some Culicidae species prey on other mosquito species that are vectors of public health importance. *Toxorhynchites (T.)*, also known as the “elephant mosquito” or “mosquito eater”, is a large, cosmopolitan genus of mosquitoes that does not consume blood [40,65,66,67]. While the adults feed on sugar-rich materials such as honeydew, fruit, and nectar, the larvae prey on the larvae of other mosquitoes as well as other nektonic (free swimming) organisms. As *Toxorhynchites* live on a protein- and fat-rich diet of aquatic organisms such as larvae, there is no requirement for blood-feeding at the adult stage, having already accumulated the necessary nutrients for oogenesis and vitellogenesis. Most species of *Toxorhynchites* live in forests, with one jungle species, *T. splendens*, consuming mosquito larvae in tree crevices (particularly those belonging to the genus *Aedes)*. *Toxorhynchites* adults are larger than *Aedes* and are considered to be harmless to humans given that they do not blood feed [39,40,67]. Taken together, these findings highlight the promising role of *Toxorhynchites* larvae as potential biocontrol agents against mosquito vectors. However, further research on the potential threat to native aquatic fauna due to the introduction of these mosquito predators is needed.

The potential of anurans (particularly frogs and toads) for mosquito control has been barely investigated [31,68,69,70]. For instance, tadpoles, with various life-history characteristics, actively prey upon the eggs of *Ae. aegypti*. It has been shown that this mosquito species has a preference to lay eggs in tadpole water and that tadpoles of *Polypedates cruciger*, as well as those of the *Bufo, Ramanella, Euphlyctis,* and *Hoplobatrachus* genera, predate on the eggs [31]. Other studies, however, have shown minimal effects with three common Thai anuran species (*Bufo melanostictus*, *Kaloula pulchra* and *Hylarana raniceps*), showing no evidence of *Cx. quinquefasciatus* larvae predation [71]. Most importantly, the biological control programs based on the release of larvivorous organisms, with special reference to amphibians and fish, are frequently not suitable in the majority of urban environments exploited by the larvae of some *Aedes* species, and require further research [13].

From an integrated vector management perspective, it has been recently observed that the employment of ultra-low quantities of botanicals or green-synthesized nanomosquitocides boosts the predation rates of a range of mosquito larvae predators. This has been demonstrated for various species of copepods (e.g., *M. edax* [58], *M. thermocyclopoides* [54], *Megacyclops formosanus* [72], *M. aspericornis* [56]), tadpoles (e.g., *Hoplobatrachus tigerinus* [70]), fish (e.g., *Gambusia affinis* [29], *Poecilia reticulata* [73], *Carassius auratus* [74], *Aplocheilus lineolatus* [21]), odonate young instars (e.g., *Anax immaculifrons* nymphs [75], *Brachydiplax sobrina* nymphs [76]), and water bugs (e.g., *Diplonychus indicus* [77]). This opportunity should be explored further, since the exploitation of synergies between ultra-low doses of plant-fabricated mosquitocides and biological control agents may represent a further control option readily available in tropical and sub-tropical developing countries worldwide [13]. 

### 2.3. Bti and Entomopathogenic Fungi

Naturally occurring organisms that are pathogenic to mosquitoes can also be considered for biocontrol strategies. *Bacillus thuringiensis* var. *israelensis* (Bti) is currently the most common mosquito larvicide employed in European countries. Bti is a gram-positive, spore-forming bacterium that releases insecticidal toxins and virulence factors that selectively target the larval stages of insects [78,79]. Application of Bti has been used to reduce the number of *Ae. aegypti* [80,81,82] and *Ae. albopictus* [83] larvae, but longer term use is subject to the development of resistance to Bti toxins [84], and the use of Bti in large mosquito breeding sites in urban environments is logistically demanding [85]. Entomopathogenic fungi produce infective spores (conidia) that attach to and penetrate the cuticle of mosquitoes, releasing toxins that result in mosquito death [86]. Several studies have shown the pathogenic effect on malaria mosquito vectors [87,88] and on *Ae. aegypti* [89,90,91]. As entomopathogenic fungi are mostly targeted towards adult mosquitoes, and because several different toxins produced during fungal infection are lethal to mosquitoes [92], selection pressure for resistance is likely to be less intense when compared to rapid-killing insecticides. Therefore, the evolution of fungus resistance is predicted to be much slower than the evolution of insecticide resistance [87]. The paucity of studies describing the effects of fungi on mosquito populations indicates further research is needed to determine the viability, infectivity, and persistence of fungal spores in mosquito field populations [93]. Clearly to deliver large-scale application of fungal spores into wild mosquito populations, optimal methods need to be determined [94].

## 3. Releasing Mosquitoes for Disease Control

### 3.1. Wolbachia Endosymbiotic Bacteria

*Wolbachia* are endosymbiotic bacteria that naturally infect approximately 40% of insect species [95] and induce a reproductive phenotype in mosquitoes known as cytoplasmic incompatibility (CI). This phenotype results in the generation of inviable offspring when an uninfected female mates with a *Wolbachia*-infected male, but *Wolbachia*-infected females can produce viable progeny when they mate with both infected and uninfected males. The overall result is a reproductive advantage for infected females, allowing this maternally transmitted bacterium to invade host populations. Natural *Wolbachia* infections are present in some major mosquito disease vectors such as *Cx. quinquefasciatus* and *Ae. albopictus*, but no natural infections are present in *Ae. aegypti*. A recent study in Burkina Faso [96] also found a novel *Wolbachia* strain in *An. gambiae s.s.* and *An. coluzzii* (major vectors of malaria in Sub-Saharan Africa). 

The first experiments to successfully use *Wolbachia* for mosquito-borne disease control utilized CI to eradicate *Cx. quinquefasciatus* mosquito populations from Myanmar in the late 1960s [97]. This incompatible insect technique (IIT) depends on releasing large numbers of *Wolbachia*-infected male mosquitoes that compete with wild type males to induce sterility and suppress the mosquito population [98]. Current targets for IIT include *Ae. albopictus* through the generation of a triple *Wolbachia*-infected strain (*w*AlbA, *w*AlbB, and *w*Pip infected) [99] and *Ae. polynesiensis*, a vector of lymphatic filariasis in the South Pacific [100]. The biotech company MosquitoMate (http://mosquitomate.com) is pioneering the use of IIT using *Ae. albopictus*, and releases of male mosquitoes are ongoing. The application of IIT is dependent on the ecology and environment which the target mosquito population inhabits. Physically isolated populations (e.g., oceanic islands) represent the optimal conditions for IIT given that large scale releases are problematic due to the need for mosquito sex separation at the pupal stage. Irradiating at the pupal stage can overcome the potential risk of unintentional fertile female release. A *Wolbachia*-infected *Ae. polynesiensis* strain that is bi-directionally incompatible with naturally infected wild type mosquitoes was irradiated at the pupal stage and this resulted in decreased fecundity and fertility in females [101]. This dose of radiation did not negatively impact male mosquito fitness parameters, mating competitiveness, or the ability to induce CI. For *Ae. albopictus*, several studies have been undertaken to determine the minimum pupal irradiation dose required to induce complete sterility in *Wolbachia* triple-infected (HC), double-infected (GUA), and uninfected (GT) female *Ae. albopictus* [102]. Irradiated *Ae. albopictus* HC, GUA, and GT strain females had decreased fecundity and fertility when irradiated and this was inversely proportional to the dose. In addition, the fitness of three *Ae. albopictus* strains (triple-infected, double-infected, and uninfected) of the same genetic background revealed that the presence of *Wolbachia* had only minimal effects on host fitness [99]. Irradiation with a female-sterilizing dose had no negative impact on the longevity of triple infected males, while a reduced lifespan was seen in wild type males (*w*AlbA and *w*AlbB) irradiated with a higher male-sterilizing dose [103]. These studies indicate that irradiation could be used to reduce the risk of unintentional release of *Wolbachia* triple-infected *Ae. albopictus* HC strain females during male release for population suppression. 

The discovery of a virulent *Wolbachia* strain in *Drosophila melanogaster* fruit flies (named *w*MelPop), which significantly lowered the lifespan of its host [104], led to further work to see if this strain could shorten the lifespan of mosquitoes. Additional *Wolbachia* strains, including the closely related avirulent *w*Mel strain, were subsequently found to protect their native hosts, *Drosophila* fruit flies, against infection by pathogenic RNA viruses [105,106]. This alternative approach for mosquito vector control relies on the use of *Wolbachia* to prevent pathogens from replicating within the mosquito [107]. The “eliminate dengue” project (www.eliminatedengue.com) based in Australia has been able to demonstrate that *Wolbachia* bacteria can prevent DENV transmission in mosquitoes without significant fitness costs. Stable *Wolbachia*-infected *Ae. aegypti* lines have now been successfully generated using embryo microinjection [108,109,110,111]. All transinfected *Wolbachia* strains significantly reduced the vector competence of *Ae. aegypti* for DENV under laboratory conditions [110,112,113]. High levels of *Wolbachia* bacteria in salivary glands was thought to be crucial to the ability to completely block DENV transmission (shown through the absence of infectious virus in the saliva) under laboratory conditions [110]. All *Wolbachia* strains showed maternal transmission rates close to 100% and induced high levels of CI in *Ae. aegypti* [108,109,110]. Semi-field cage experiments were undertaken to assess fitness costs and the ability of two *Wolbachia* strains to invade mosquito populations. The fecundity of *w*MelPop-infected female mosquitoes was reduced by ~60% relative to uninfected wildtype and *w*Mel-infected mosquitoes, and this strain invaded at a slower rate when compared to *w*Mel [110]. Mosquitoes infected with the *w*Mel strain were introduced into the wild through open releases at two locations near Cairns in north Queensland, Australia, and reached near fixation within a few months [114].

The success of these preliminary field releases has led to subsequent releases in Australia and now countries that experience high dengue cases such as Indonesia, Vietnam, Colombia, and Brazil (www.eliminatedengue.com). One potential concern for a *Wolbachia*-replacement approach is the future development of resistance to *Wolbachia*’s inhibitory effects. Although no studies to date have demonstrated that this is likely to happen, a *Wolbachia*-superinfected line was recently established in *Ae. aegypti* containing stable infections of the *w*Mel and *w*AlbB strains that could help mitigate potential resistance. This combination of strains resulted in greater inhibitory effects on DENV replication than the single *w*Mel strain when challenged with blood meals from viraemic dengue patients [111]. *Wolbachia* superinfections could be utilised to replace single infections in wild populations and could help overcome any resistance by DENV to singly infected strains that are present in wild mosquito populations.

As only preliminary trials are underway for this promising strategy, a number of questions remain regarding implementation in the field. The applied use of *Wolbachia* for dengue control needs further research to determine the best individual or combination of *Wolbachia* strains. This has to take into account both the effects on DENV transmission and any resulting mosquito fitness costs. To predict the impact of the *w*Mel strain would have on dengue transmission, mathematical models were produced to show that a 66%*–*75% reduction in the basic reproductive number, R_0_, could be achieved [115]. Ultimately, further experiments are needed to determine the overall effect *Wolbachia* will have on DENV transmission and dengue epidemiology in the field, particularly in endemic areas. Finally, *Wolbachia*-infected *Ae. aegypti* could also play a role in reducing transmission of other mosquito-borne diseases, as *Wolbachia* inhibits the transmission of chikungunya virus (CHIKV) [112,116], yellow fever virus (YFV) [117], malaria parasites [118,119], and Zika virus (ZIKV) [120]. Given the recent outbreaks of ZIKV in South America, novel control strategies including *Wolbachia* should be considered if *Ae. aegypti* is responsible for outbreaks in the Americas [14]. Another arbovirus, Japanese encephalitis virus (JEV), is transmitted mostly by *Cx. tritaeniorhynchus* mosquitoes and the epidemiology of this zoonotic disease would suggest *Wolbachia* could also reduce transmission provided stable transinfection is achieved [15]. Additional mosquito species, such as *Cx. quinquefasciatus* and *Ae. albopictus* that contain resident *Wolbachia* strains, are also potential targets for introducing “transinfected” strains that are likely to grow to higher densities and therefore impact pathogen transmission [121].

### 3.2. The Sterile Insect Technique

The Sterile Insect Technique (SIT) is a genetic suppression strategy that involves rearing large numbers of males of the target species and either irradiating or treating them with chemosterilizing agents to generate chromosomal aberrations and dominant lethal mutations in sperm. These sterilized male insects are released and when they mate with wild females produce no progeny. A sustained SIT programme results in an increasing ratio of released sterile males to wild males (as the population decreases) eventually leading to population elimination. Major interventions over the past 50 years using SIT against agricultural pests have proved very successful, including the eradication of the New World screwworm, *Cochliomyia hominivorax*, from North and Central America, and the eradication of *Glossina austeni* tsetse flies from Unguja Island, Zanzibar [122]. The use of SIT for mosquitoes that transmit human disease has been limited due to the reduced performance of sterilized males caused by sterilization. An additional problem for SIT programmes (and any other mosquito suppression strategy that aims for eradication) targeted towards *Aedes* species is the difficulty of the initial need to reduce the wild population densities, prior to the release of sterile males [123].

In addition to the IIT approaches using *Wolbachia* in combination with female sterility, renewed interest in SIT for the suppression of mosquito vectors has come through experiments to combine SIT with other forms of delivering mosquito lethality [124]. SIT combined with auto-dissemination, in which adult females are contaminated with dissemination stations of juvenile hormone (e.g., pyriproxygen), could be used to treat breeding habitats of *Ae. aegypti* and *Ae. albopictus* [125]. Contaminated female mosquitoes would lay eggs in larval sites and the insect growth regulator, introduced to the aquatic environment by the female, would prevent adult mosquito eclosion. Successful suppression using juvenile hormones was achieved for both *Ae. aegypti* in Peru [123] and *Ae. albopictus* in Spain [125]. Releasing sterile males with a juvenile hormone, such as pyriproxygen, could also allow contamination of females during mating to “boost SIT” [126]. SIT could also be enhanced by using sterile males to deliver densoviruses to their wild counterparts [126]. A European project entitled “Revolutionizing insect control” has recently started to determine if dispersion of mosquito densoviruses (MDVs), species-specific natural entomopathogenic viruses, by sterile males to wild females results in detrimental effects on *Ae. aegypti* and *Ae. albopictus* larval habitats, as a result of their skipping oviposition behaviour [127]. MDVs replicate in the nuclei of mosquito cells and kill mosquito larvae in a dose-dependent manner. Larvae that survive do not pupate or eclose to adults, resulting in an overall reduction in the mosquito population. As a result, MDVs have been proposed as potential biocontrol agents as they are also highly specific to target mosquito species. Female mosquitoes infected with an MDV can transmit the virus vertically to their progeny suggesting MDVs could persist and spread through wild mosquito populations. Laboratory studies using MDVs have shown high rates (>80%) of *Ae. aegypti* larval mortality [128]. However, the efficacy and sustainability of MDVs as a biocontrol agent was tested in and among oviposition sites in large laboratory cages, but was not shown to significantly reduce *Ae. aegypti* egg densities [129]. A direct inhibitory effect of MDVs on arboviral replication in cell lines has also been shown [130,131,132] which could work synergistically with pathogenic effects on the mosquito vector. However, co-infection of MDV and CHIKV in adult *Ae. aegypti* mosquitoes [133] suggests that MDVs may not be effective against all medically important arboviruses.

### 3.3. Genetically Modified Mosquitoes

An alternative method to sterilise males for insect population suppression has been developed in which a self-limiting gene is introduced into mosquito populations through genetic engineering [134]. This approach, pioneered by the British biotech company Oxitec (www.oxitec.com), was named Release of Insects carrying a Dominant Lethal (RIDL). The lethal gene can be repressed using an antidote (tetracycline) so that mosquitoes can be reared to adulthood in rearing facilities prior to the release of males into wild populations, which then mate with wild females, producing offspring that die at the larval stage in the absence of tetracycline. This approach has the advantage of being species-specific (like IIT and SIT) and has no long lasting effects on the target species as the aim is to eliminate the population in the release area. Field trials in the Cayman Islands in 2009–2010 with a self-limiting strain of *Ae. aegypti* OX513A were shown to suppress a wild population of *Ae. aegypti* [135]. In Malaysia, OX513A males were shown to have similar longevity and dispersal capabilities [136] and the latest release of OX513A males in Brazil led to strong suppression of the target wild population [137]. Trials in Brazil using RIDL male releases were conducted in a small suburb of Juazeiro, Bahia, and larger trials will be required to determine if the observed level of local suppression can be scaled up to larger release areas. RIDL technology was also used to generate a strain of *Ae. aegypti*, LA513A, engineered to carry a dominant, repressible, non-sex-specific, late-acting lethal genetic system, resulting in death at the pupal, rather than larval stage to avoid density dependent effects on larval development in wild populations [138]. In the absence of tetracycline, larvae carrying one or more copies of the LA513A insertion develop normally but the vast majority (95%–97%) die at pupation [138]. This incomplete penetrance of the lethal phenotype, however, could potentially result in unknown environmental consequences given this strategy is reliant on a self-limiting strain of mosquitoes.

Another potential method to suppress or eliminate mosquito populations is to induce an extreme male-biased sex ratio [139]. Although naturally occurring sex ratio distorters were found in *Aedes* and *Culex* mosquitoes, population suppression was not achieved in cage experiments [140]. Genetic modification can provide a bias towards male gamete production by inducing preferential breakdown of the X chromosome during male meiosis. Breakdown of the paternal X chromosome in *An. gambiae* prevents it from being transmitted to the next generation, resulting in fully fertile mosquito strains that produce >95% male offspring [141]. These synthetic distorter male mosquitoes suppress caged wild type mosquito populations, providing evidence for potential new strategies for mosquito vector control. It must be noted that Culicine mosquitoes contain homomorphic sex chromosomes (containing only a small nonrecombining region) [142] which may limit this approach for major Culicine mosquito vectors.

## 4. Behavioural Knowledge: A Tool to Enhance Mosquito Control Programs?

### 4.1. Behavioural Quantification Helps SIT

Research into understanding the basics of mosquito mating ecology (particularly sexual chemical ecology) has been limited in the context of informing vector control strategies [13,143]. If greater information is obtained on the mating behaviour of medically important mosquito species, it could enhance control programs. For example, a crucial factor in the success of SIT, IIT, and RIDL progams is the ability of sterile male mosquitoes to compete with wild type males when mating with females [124,144,145]. Greater knowledge of mosquito swarming and mating behaviour could be used to compare courtship and mating ethograms of different mosquito vector species. The parameters underlying male mating success can be used to inform control programs. For example, the age, body size, and density in swarms of male *Ae. aegypti* can influence mating success [146,147], in addition to the role of mosquito mating acoustics in *Ae. aegypti* field swarms [148]. Further information on the parameters underlying male mating success could then be used to inform control programs to generate high quality mass-released males (in the case of SIT, boosted SIT, IIT, and RIDL) and to monitor the mating performance of *Wolbachia*-infected males [13,149]. It is worth noting that quantitative analyses of mating ethograms in mosquitoes are rare, and mainly focus on the elaborate courtships found in the genera *Sabethes* [150,151,152] and *Wyeomyia* [153]. The majority of studies investigating the sexual behaviour of medically important *Aedes* species just compare the insemination ability in sterilised and wild type males [154,155,156,157,158,159,160,161]. Behavioural quantification of courtship and mating events has often been excluded in sexual behaviour studies [149,162,163]. Notably, there is also only limited information on the molecules that mediate mosquito aggregation and mating [164,165].

### 4.2. Sound Traps

Vector control stratagies incorporating sound traps were first attempted in Cuba in 1949 against *An. albimanus*, in which sound traps were used to collect an elevated number of male mosquitoes [166]. Further trials using sound traps resulted in the trapping of *Cx. tarsalis* males, leading to a reduction in insemination of females [167] and reductions in the number of *Cx. tritaeniorhynchus* parous females [168]. Sound traps rely on wing-beat frequencies which can overlap for different mosquito species (potentially attracting multiple vector species) [169,170]. However, field trials with sound traps have shown limited success for several reasons. Firstly, there are technical difficulties in designing a sound trap that has the required amplification that will attract mosquitoes from long distances. The location in which the traps are placed also needs consideration, with improved efficacy for close proximity to swarming sites. Locating swarming sites, particularly for *Anopheles* species, needs significant development if sound traps are to be used for mosquito control [169].

### 4.3. The “Lure and Kill” Technique

The “lure and kill” approach has been successful for several arthropod pest species [171] and has been proposed to have a potential role for the control of mosquitoes (particularly *Anopheles* species) [169]. For *Anopheles* mosquitoes, visual stimuli are thought to be important in the convergence of individuals to a swarming site [172,173]. Consequently, recent research has revealed the potential to disrupt or enhance swarms, through manipulation of artificial swarm markers (or landmarks). This could lead to the development of “kill zones” that kill large numbers of attracted mosquitoes. In order for this to be applicable in field settings, rapid and economical methods to locate swarming sites need to be developed [143,169].

## 5. Conclusions and Future Perspectives

Biocontrol strategies for mosquito-borne diseases are needed to help reduce the prolonged application of insecticides that are currently used as the primary method for mosquito control. Eco-friendly, safe, and sustainable methods should be developed that can target a range of different mosquito species. Mosquito predators can be very effective in certain conditions, as demonstrated by the elimination of *Ae. aegypti* populations in rural Vietnam. The pathogenic bacterium Bti has been extensively used due to its ability to selectively kill mosquito larvae, and additional pathogens, such as entomopathogenic fungi, may be effective in future control programs. One of the most promising novel strategies is the use of *Wolbachia* endosymbiotic bacteria, which has been targeted towards reducing DENV transmission. Despite significant progress so far, larger scale trials are needed to determine if *Wolbachia*-based strategies can be an effective method of mosquito biocontrol. A combination of synergistic strategies may be required for effective population suppression using methods such as SIT, RIDL, and *Wolbachia*-induced IIT [144]. Mosquito behaviour plays a key role in vector control programs and further knowledge regarding the chemical ecology of mate searching, swarming landmarks, and mate choice in swarming sites is required to improve control strategies.
